# Transjugular Intrahepatic Portosystemic Shunt Is Associated With Better Waitlist Management of Liver Transplant Candidates With Hepatocellular Carcinoma

**DOI:** 10.3389/ti.2024.12781

**Published:** 2024-06-26

**Authors:** Sofia El Hajji, Stéphanie Lacotte, Beat Moeckli, François Cauchy, Philippe Compagnon, Christian Toso

**Affiliations:** ^1^ Division of Abdominal Surgery, Department of Surgery, Geneva University Hospitals, Geneva, Switzerland; ^2^ Laboratory of Transplantation and Hepatology, University of Geneva, Geneva, Switzerland; ^3^ Division of Transplantation, Department of Surgery, Geneva University Hospitals, Geneva, Switzerland

**Keywords:** liver transplantation, recurrence, survival, progression, hepatocellular carcinoma

## Abstract

Transjugular intrahepatic portosystemic shunt (TIPS) reduces portal hypertension complications. Its impact on hepatocellular carcinoma (HCC) remains unclear. We evaluated 42,843 liver transplant candidates with HCC from the Scientific Registry of Transplant Recipients (2002–2022). 4,484 patients with and without TIPS were propensity score-matched 1:3. Analysing wait-list changes in total tumor volume, HCC count, and alpha-fetoprotein levels, and assessing survival from listing and transplantation; TIPS correlated with a decreased nodule count (−0.24 vs. 0.04, *p* = 0.028) over a median wait period of 284 days (IQR 195–493) and better overall survival from listing (95.6% vs. 91.5% at 1 year, *p* < 0.0001). It was not associated with changes in tumor volume (0.28 vs. 0.11 cm³/month, *p* = 0.58) and AFP (14.37 vs. 20.67 ng/mL, *p* = 0.42). Post-transplant survival rates (91.8% vs. 91.7% at 1 year, *p* = 0.25) and HCC recurrence (5.1% vs. 5.9% at 5 years, *p* = 0.14) were similar, with a median follow-up of 4.98 years (IQR 2.5–8.08). While TIPS was associated with a reduced nodule count and improved waitlist survival, it did not significantly impact HCC growth or aggressiveness. These findings suggest potential benefits of TIPS in HCC management, but further studies need to confirm TIPS safety.

## Graphical Abstract

**Figure:**
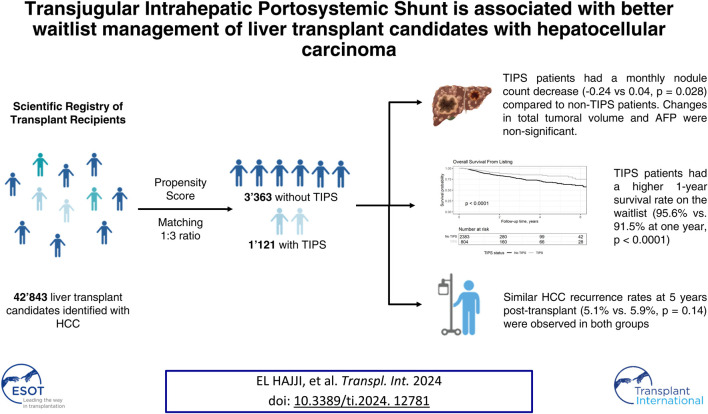
Created with Biorender.com.

## Introduction

Transjugular intrahepatic portosystemic shunt (TIPS) is a valuable interventional strategy to alleviate portal hypertension complications. It effectively diverts blood flow from the portal vein to the hepatic veins, lowering portal pressure and its subsequent clinical manifestations including ascites and variceal bleeding [1, 2]. Despite its clinical advantages in portal hypertension, the role of TIPS in the management of patients remains unclear [3, 4]. Some authors have revealed no association between TIPS and *de novo* HCC nodules, while others caution against a potentially increased risk of HCC occurrence [5–8]. Concerns regarding the potential influence on tumor behavior persist, especially considering the limited sample sizes of many studies. Alterations in hepatic blood flow dynamics could theoretically promote tumor growth or metastasis through various mechanisms, including hypoxic liver injury, dissemination at insertion, or reduced response to locoregional treatment [9, 10].

We took advantage of a large prospective database from the Scientific Registry of Transplant Recipients (SRTR), which includes mandatory data from all liver transplant candidates in the United States. While on the list, HCC patients undergo periodic imaging and alpha-fetoprotein (AFP) assessments to benefit from exception Model for End-Stage Liver Disease (MELD) points. This dataset granted us access to data on HCC characteristics, such as size, number, and AFP, while patients were on the waitlist. Our study focused on comparing patients with and without TIPS at the time of listing to elucidate its impact on the progression of HCC.

## Materials and Methods

### Study Population

This study utilized data from the SRTR database, a prospective registry that contains information on all donors, wait-listed candidates, and transplant recipients in the United States. The SRTR registry, submitted by members of the Organ Procurement and Transplantation Network (OPTN), encompasses a comprehensive list of patients registered from February 01, 2002, which corresponds to MELD implementation in the United States, to June 2, 2022, date of data retrieving.

Our study selected patients diagnosed with HCC as their primary or secondary diagnosis and compared them with (1,132) versus without TIPS (21,393) at the time of listing. Patients with liver tumors other than HCC were also excluded from the study. The TIPS status was determined prior to listing using the “CAN_TIPSS” label. We aimed to investigate the variations in HCC characteristics among patients on the waiting list for transplantation.

### Data Collection

Data management and analysis were conducted using the R studio software (version 2022.07.2 + 576) [11]. Patient characteristics included age, sex, body mass index (BMI), underlying liver disease diagnosis, date of listing, date of transplantation, date of death, and time of follow-up. We classified the underlying liver diseases as viral, non-alcoholic steatohepatitis (NASH), and alcoholic liver disease (OH). An “other” category encompassing less prevalent etiologies like metabolic disease, cholestatic disease, drug exposure, and autoimmune disorders, each constituting less than 5% of the studied population. MELD was calculated in accordance with the 2016 revision by the United Network for Organ Sharing using a custom R function that assigned a minimum value of 1 to any log-scaled values less than 1 to prevent negative scores. Sodium levels were capped between 125 and 137 mg/dL, whereas creatinine levels were capped at 4 mmol/L. The maximum attainable MELD score was 40.

We collected HCC characteristics at each MELD exception update from the “MPEXCEPT” list, allowing longitudinal monitoring of each patient. The characteristics included the HCC diameter, count, and AFP levels. For patients with multiple HCCs, the total tumor volume (TTV) was calculated by summing the volumes (calculated as the volume of a sphere V = 4/3πr³) of the individual HCCs. The tumor burden was also evaluated based on the number of tumors. We assessed changes in TTV and tumor count between the first (at listing) and last (or pre-transplant) assessments, measuring changes per patient in volume in cm³ per month and count in units per year. Changes in AFP levels were expressed in ng/mL per month. In terms of therapeutic interventions, HCC treatments were categorized as: “curative” when cryotherapy, thermoablation, chemical ablation, or surgery were used; “locoregional chemotherapy” when chemoembolization was used; “mixed” when both modalities were used; or “untreated” in the absence of HCC-directed treatment.

### Propensity Score Matching

Propensity score matching (PSM) was performed using the “MatchIt” package to achieve covariate balance and mitigate selection bias between groups with and without TIPS [12]. Prior to performing the matching, we ensured that only patients with complete data for the matching criteria and their outcomes were evaluated. Matching utilized nearest-neighbor matching with a 3:1 pairing ratio to optimize the analysis. Patients were matched based on age, body mass index (BMI), underlying liver disease, initial calculated TTV, nodule count, AFP levels, waitlist HCC treatment category, and the calculated MELD score. The aim of this study was to minimize differences in liver function and initial HCC characteristics between the TIPS and non-TIPS groups to better capture the effect of TIPS on HCC, including TTV, nodule count, and AFP levels.

### Statistical Analysis

Survival was first evaluated from listing by censoring transplanted patients in the matched cohort. Post-transplant survival was then studied in patients who eventually underwent transplantation from the matched cohort. We used the listing date, transplantation date, and death date to compute the survival curves. Post-transplant HCC recurrence was determined following a procedure previously used by our group and others in the same cohort [13, 14]. Notably, this procedure provides an accurate assessment of recurrence rate.

Statistical analyses were conducted using the R Studio software. The analytical results were visualized using the “gtsummary” package [15]. To compare sample distributions, we employed the Welch two-sample *t*-test, Wilcoxon test, and Pearson’s chi-squared test. For survival analysis, we utilized both the “survival” and “survminer” packages [16, 17]. The Kaplan-Meier method was used to assess overall survival (OS), and differences between groups were assessed using the log-rank test. The cumulative incidence risk of HCC was calculated using the “tidycmprsk” package, and the differences were compared using Gray’s test [18]. Statistical significance was set at a threshold of *p* < 0.05.

## Results

### Demographics

During the study period (data dating back from February 1, 2002, until June 2, 2022), a total of 42,843 patients diagnosed with HCC were placed on the waiting list. Patient characteristics are reported in Table 1, and the measured outcomes of HCC progression are shown in Table 2. Patients with TIPS were younger, had a higher BMI, and had a higher prevalence of alcohol-related liver disease. These patients also displayed more advanced liver disease, as indicated by higher Model for MELD scores, but less advanced HCC staging as shown by their TTV et number of tumors at listing.

**TABLE 1:** Demographics of the selected HCC patients compared between patients with (TIPS) and without (No TIPS) a history of TIPS.

Demographics	No TIPS, N = 40,691	TIPS, N = 2,152	*p*-value^a^
Age at listing (years), Mean (SD)	59.61 (7.9)	58.97 (7.7)	<0.001
Gender, n (%)			0.15
F	9,565 (24)	477 (22)	
M	31,126 (76)	1,675 (78)	
Body mass index (kg/m^2^), Mean (SD)	28.91 (5.4)	29.60 (5.6)	<0.001
Underlying liver disease, n (%)			
Hepatitis B	1,497 (3.7)	43 (2.0)	
Hepatitis C	12,958 (32)	570 (26)	
Hepatitis C and B	180 (0.4)	6 (0.3)	
Hepatitis viral other	16 (<0.1)	1 (<0.1)	
NASH	3,348 (8.2)	249 (12)	
OH	4,814 (12)	498 (23)	
Other	17,878 (44)	785 (36)	
Last calculated MELD score, Mean (SD)	14.08 (7.6)	16.60 (7.1)	<0.001
Unknown	1,140	73	
Waitlist HCC treatment, n (%)			<0.001
curative	3,166 (7.8)	164 (7.6)	
locoregional	20,390 (50)	953 (44)	
mixed	2,332 (5.7)	75 (3.5)	
untreated	14,803 (36)	960 (45)	

^a^ Welch Two Sample *t*-test; Pearson’s Chi-squared test.

**TABLE 2:** Outcomes on HCC evolution measured on the whole cohort and compared between patients with and without TIPS.

Outcomes	No TIPS, N = 40,691	TIPS, N = 2,152	*p*-value^a^
TTV at listing (cm³), Mean (SD)	17.16 (243.1)	13.34 (16.1)	0.004
*Unknown*	*4,653*	*238*	
TTV change (cm³/month), Mean (SD)	−0.28 (10.9)	−0.04 (12.5)	0.51
*Unknown*	*17,699*	*952*	
Number of tumors at listing, Mean (SD)	1.30 (0.6)	1.27 (0.6)	0.030
Number of tumors change (unit/year), Mean (SD)	−0.04 (6.7)	−0.21 (1.7)	0.010
*Unknown*	*15,813*	*861*	
AFP at listing (ng/mL), Mean (SD)	140.80 (1,230.5)	119.36 (1,002.3)	0.34
*Unknown*	*811*	*42*	
AFP change (ng/mL per month), Mean (SD)	−14.78 (3,293.3)	13.29 (167.0)	0.18
*Unknown*	*14,520*	*815*	

^a^ Welch Two Sample *t*-test.

Italicized data are missing values.

### Propensity Score Matching

Considering the disparities between the groups, we implemented propensity score matching to equilibrate the data. This approach allowed us to investigate the specific effects of TIPS on HCC volume, number, and AFP changes over a median waiting time 284 days (IQR 195–493). The matching process was performed on a 3:1 basis and accounted for the covariates described in the Methods section. The balanced data are presented in Table 3.

**TABLE 3:** Balanced table of the matched cohort.

Demographics	No TIPS, N = 3,363	TIPS, N = 1,121	*p*-value^a^
Age at listing (years), Mean (SD)	59.26 (8.1)	59.34 (7.7)	0.78
Gender, n (%)			0.65
F	839 (25)	272 (24)	
M	2,524 (75)	849 (76)	
Body mass index (kg/m^2^), Mean (SD)	29.96 (5.7)	29.79 (5.6)	0.38
Underlying liver disease, n (%)			
Hepatitis B	36 (1.1)	20 (1.8)	
Hepatitis C	878 (26)	289 (26)	
Hepatitis C and B	2 (<0.1)	2 (0.2)	
NASH	447 (13)	134 (12)	
OH	690 (21)	252 (22)	
Other	1,310 (39)	424 (38)	
Last calculated MELD score, Mean (SD)	16.17 (8.2)	16.23 (6.7)	0.78
Waitlist HCC treatment, n (%)			0.77
curative	260 (7.7)	84 (7.5)	
locoregional	1,836 (55)	612 (55)	
mixed	138 (4.1)	54 (4.8)	
untreated	1,129 (34)	371 (33)	

^a^ Welch Two Sample *t*-test; Pearson’s Chi-squared test.

### HCC-Related Data

Following propensity matching, the HCC characteristics between patients with and without TIPS did not reach statistical significance anymore, as outlined in Table 4, this was done to match the patients on tumor biology as closely as possible. We then explored the waitlist changes to capture the effect of TIPS on HCC progression. A negative change in HCC volume or count indicates an effective tumor treatment or resection. Conversely, a positive monthly change was indicative of ineffective treatment (or absence of treatment) and/or more aggressive HCC.

**TABLE 4:** Outcomes on HCC evolution after matching.

Outcomes	No TIPS, N = 3,363	TIPS, N = 1,121	*p*-value^a^
TTV at listing (cm³), Mean (SD)	12.23 (18.0)	12.70 (16.1)	0.41
TTV change (cm³/month), Mean (SD)	0.11 (13.2)	0.28 (6.5)	0.58
Number of tumors at listing, Mean (SD)	1.27 (0.5)	1.28 (0.6)	0.60
Number of tumors change (unit/year), Mean (SD)	0.04 (6.5)	−0.24 (1.9)	0.028
AFP at listing (ng/mL), Mean (SD)	55.38 (262.5)	56.27 (334.3)	0.94
AFP change (ng/mL per month), Mean (SD)	20.67 (328.3)	14.37 (177.9)	0.42

^a^ Welch Two Sample *t*-test.

TIPS was associated with a decrease in the number of HCC, potentially indicating more efficient treatment of these lesions. There were no significant changes in volume or AFP dynamics between the groups as presented in Table 4.

Of note, we also performed a sensitivity analysis, also including patients with missing data. Similar outcomes have been observed, with a decrease in the number of HCC, and no change in volume and AFP dynamics (data not shown).

### Overall Survival From Listing

**FIGURE 1:**
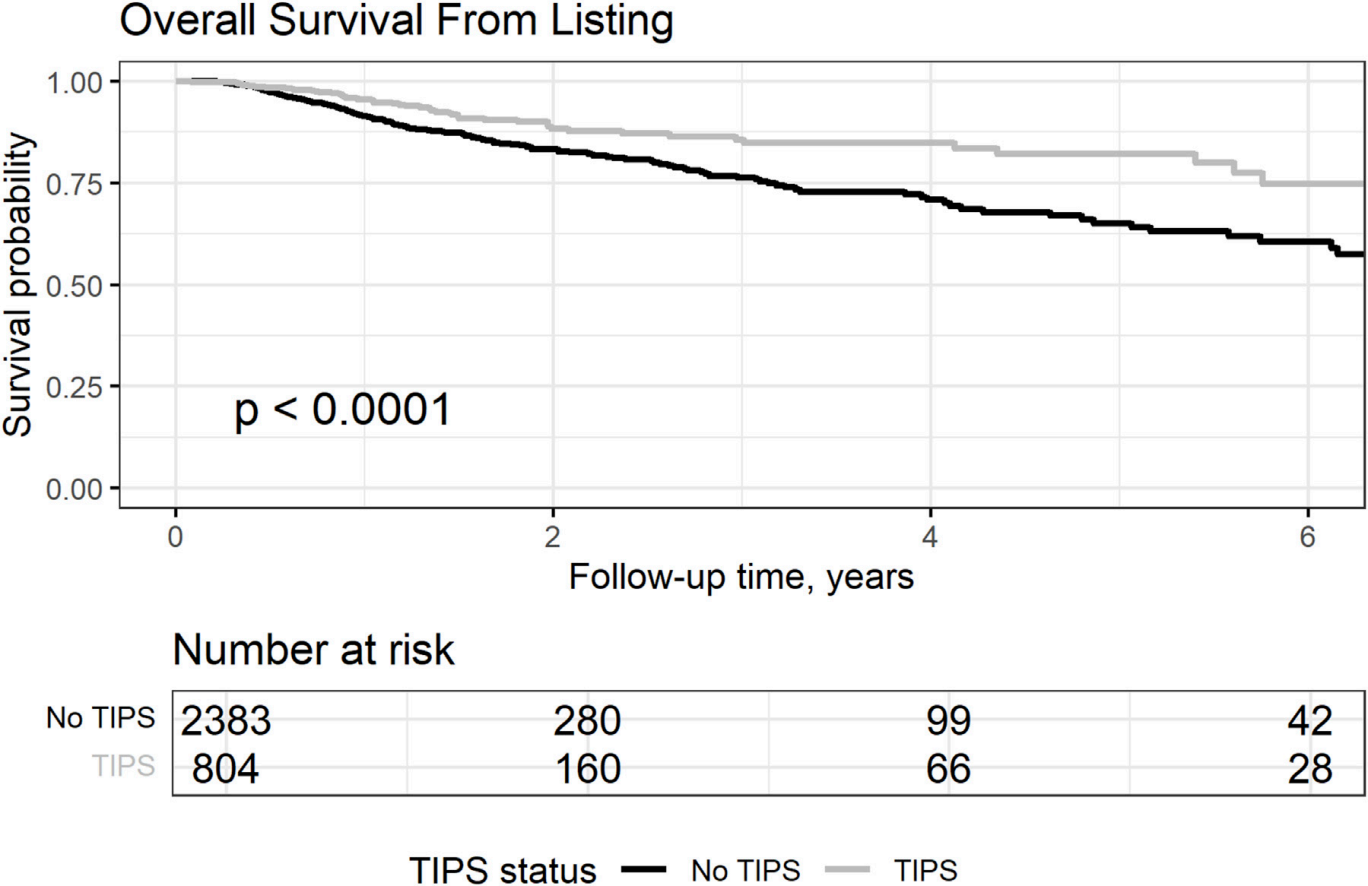
Overall survival from listing in the matched cohort, censoring transplanted patients and comparing patients with and without TIPS.

We compared overall survival (OS) from listing between patients with and without TIPS in the matched cohort. OS at 1, 5, and 10 years accounted for 95.6%, 82.1%, and 66%, respectively, in the TIPS and 91.5%, 65.1%, and 52%, respectively, in the no-TIPS group (log-rank test: *p* < 0.0001), as shown in Figure 1. Despite a longer waiting time to transplant for the TIPS group, which was 324 days (IQR 210; 607) compared to 272 days (IQR 191; 463) for the non-TIPS group (Wilcoxon test *p* < 0.001), survival rates were notably higher in the TIPS group.

We further explored the causes of the observed differences in the survival rates. Removal from the waitlist concerned 26% (N = 268) of TIPS and 24% (N = 888) of non-TIPS patients. Among them, 50% (N = 135) of TIPS vs. 46% (N = 408) of non-TIPS patients were too ill to be transplanted, and 23% (N = 56) of TIPS vs. 29% (N = 224) of non-TIPS patients died. When exploring the causes of death, hemorrhage-related death was more frequent in the non-TIPS group (1.8%, N = 1 in TIPS and 7.6%, N = 17 in non-TIPS patients).

### Post-Transplantation Outcomes

**FIGURE 2:**
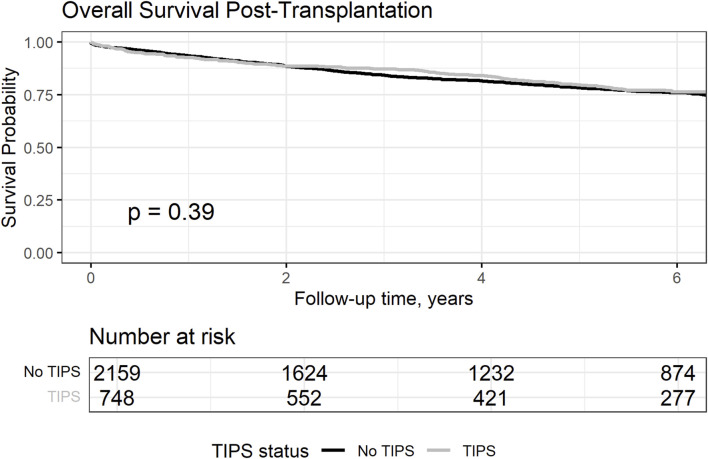
Overall survival from transplantation in the matched cohort comparing patient with and without TIPS.

Analysis of post-transplant overall survival rates revealed no statistically significant difference between patients with and without TIPS (Figure 2). At 1, 5, and 10 years post-transplant, survival rates were comparable between the TIPS (92.6%, 79.6%, and 68.8%, respectively) and no-TIPS group (93.4%, 78.3%, and 67.1%, respectively, *p* = 0.39). The time of follow-up from listing was also similar (5.14 versus 4.88 years, *p* = 0.14). These results suggest that TIPS does not affect post-transplantation survival in patients with HCC, which aligns with previous data [19, 20].

**FIGURE 3:**
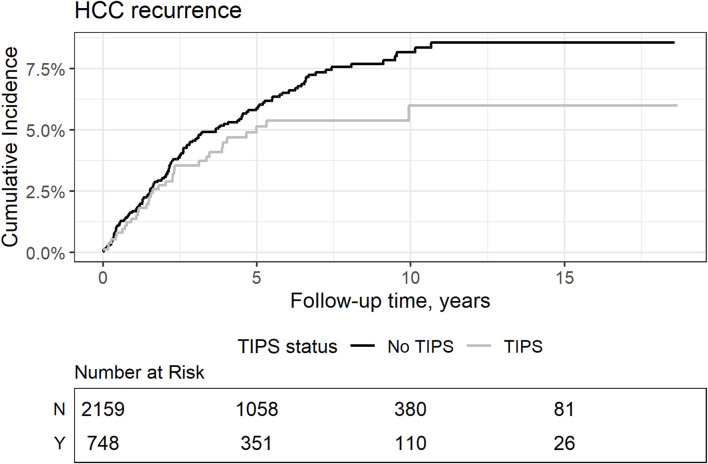
Competed cumulative risk incidence of HCC recurrence comparing patients with and without TIPS. Gray’s test *p* = 0.14.

Considering the observed HCC dynamics while on the waitlist, we further explored whether this could have an impact on the risk of posttransplant HCC recurrence (Figure 3). The cumulative risk incidence of post-transplant HCC recurrence at 5 years was similar between the groups (5.1% vs. 5.9% without TIPS, Gray’s test, *p* = 0.14).

## Discussion

Our study contributes to the growing body of literature that explores the potential impact of TIPS on HCC. Utilizing a large patient cohort, we present novel insights into the specific advantages conferred by TIPS, especially in the context of tumor burden and survival dynamics among patients awaiting transplantation. A significant finding in our study is that patients with TIPS not only exhibited improved survival while on the waiting list, but also a reduction in the number of HCC nodules. Furthermore, TIPS was not associated with a significant impact on HCC volume or AFP changes.

In line with previous studies [19-24] our findings highlight the benefits of TIPS placement on survival outcomes. This effect may stem from its efficacy in alleviating portal hypertension, enabling concurrent treatment, or reducing bleeding events, potentially serving as a bridge to liver transplantation [22, 25]. Conversely, in cases of advanced HCC, other studies have found that TIPS significantly improved OS by reducing bleeding episodes [26]. However, when assessing TACE efficacy specifically in HCC patients with TIPS, Kuo et al. [10] observed reduced efficacy and shorter overall survival (OS) in the TIPS group. A full understanding of how TIPS influences HCC behavior and treatment response requires further cellular-level investigations that may help establish a conclusive link between TIPS placement and enhanced overall survival.

Prediction models have been developed to examine HCC recurrence after liver transplantation, focusing on factors such as nodule count, size, AFP levels, and vascular invasion, among others [27, 28]. Although the effect of TIPS on posttransplant recurrence has not been extensively explored, our study highlights that TIPS does not affect the risk of HCC recurrence.

Consistent with our results, a meta-analysis of 859 patients by Chen et al. [6] reported that TIPS placement did not increase the risk of HCC development among patients with cirrhosis. This might be due to the reduced proliferative activity of hepatocytes observed after TIPS placement, as reported by Delhaye et al. [29] In contrast, two different studies investigated the impact of TIPS on hepatic blood flow [30, 31] noted increased hepatic blood flow, particularly during the arterial phase of imaging. This observation raises concerns about potential HCC growth subsequent to arterialization of the liver. However, to our knowledge, a direct correlation between TIPS placement and HCC growth has not been established.

The significant difference in OS between the TIPS and non-TIPS groups is noteworthy. This highlights the effectiveness of TIPS as a bridging therapy to enhance life expectancy even in the presence of HCC. The decrease in hemorrhage-related deaths in the TIPS group further supports this notion, indicating the role of this procedure in mitigating the risks associated with portal hypertension.

The precise mechanisms by which TIPS modifies the liver parenchyma and HCC dynamics remain only partially understood. Further histopathological investigations should be performed to understand how TIPS modifies liver vascularization, enabling a more comprehensive treatment strategy for these patients.

Although our study employed propensity score matching, the potential for unmeasured confounders remains a limitation. Moreover, the presence of missing data in our analysis indicates the need for more comprehensive data collection in future studies, including the date of TIPS placement and its correlation with HCC appearance, which could offer insights into the immediate complications of the procedure and potential cancer dissemination in cases of misplacement. Eventually, we acknowledge the heterogenous nature of the SRTR dataset and the potential bias introduced by varying levels of experience and expertise across different centers. Experienced interventional radiology teams could indeed influence the outcomes observed in the TIPS group and future analyses should be include this confounding factor. Future prospective studies are required to validate our findings and to further elucidate the nuanced effects of TIPS on HCC behavior.

In conclusion, our findings support the general beneficial use of TIPS in HCC patients. Although the procedure may stabilize or decrease new tumor formation, it appears that it does not affect HCC growth according to our analyses. Coupled with the observed reduction in hemorrhage-related deaths and improved overall survival, TIPS has emerged as an efficient intervention, particularly for patients awaiting liver transplantation. However, establishing the definitive benefits and risks of TIPS in these patients should be accomplished in future prospective studies.

## Data Availability

The data analyzed in this study is subject to the following licenses/restrictions: The data extracted from the SRTR research database is maintained by HHRI solely for the use of the author. The recipient of released data will abide by the terms stated in the Agreement Clauses. Requests to access these datasets should be directed to sofia.elhaijji@hcuge.ch.

## Abbreviations

AFP: alpha fetoprotein; BMI: body mass index; OS: overall survival; HCC: hepatocellular carcinoma; MELD: Model for End-Stage Liver Disease; SRTR: Scientific Registry of Transplant Recipients; TIPS: transjugular intrahepatic portosystemic shunt; TTV: total tumoral volume.
